# A multicenter prospective study of next-generation sequencing-based newborn screening for monogenic genetic diseases in China

**DOI:** 10.1007/s12519-022-00670-x

**Published:** 2023-02-27

**Authors:** Ru-Lai Yang, Gu-Ling Qian, Ding-Wen Wu, Jing-Kun Miao, Xue Yang, Ben-Qing Wu, Ya-Qiong Yan, Hai-Bo Li, Xin-Mei Mao, Jun He, Huan Shen, Hui Zou, Shu-Yuan Xue, Xiao-Ze Li, Ting-Ting Niu, Rui Xiao, Zheng-Yan Zhao

**Affiliations:** 1 National Clinical Research Center for Child Health, Children’s Hospital, Zhejiang University School of Medicine, Hangzhou, China; 2 Chongqing Health Center for Women and Children, Neonatal Screening Center, Chongqing, China; 3 Guiyang Maternal and Child Health Hospital, Guiyang, China; 4 University of the Chinese Academy of Science, Shenzhen Hospital, Shenzhen, 518000 Guangdong China; 5 Shanxi Children’s Hospital Shanxi Maternal and Child Health Hospital, Taiyuan, Shanxi China; 6 The Central Laboratory of Birth Defects Prevention and Control, Ningbo Women and Children’s Hospital, Ningbo, 315012 Zhejiang China; 7 Maternal and Child Health Hospital of Ningxia Hui Autonomous Region, Yinchuan, China; 8 Changsha Maternal and Child Health Hospital, Changsha, Hunan China; 9 Yunnan Maternal and Child Health Hospital, Kunming, Yunan China; 10 Jinan Maternity and Child Care Hospital Affiliated to Shandong First Medical University, Jinan, Shandong China; 11 Urumqi Maternal and Child Health Care Hospital, Xinjiang Uygur Autonomous Region, Urumqi City, China; 12 Medical Genetic Center, Changzhi Maternal and Child Health Care Hospital, Changzhi, Shanxi China; 13 Maternal and Child Health Care Hospital of Shandong Province, Jinan, Shandong China; 14 National Engineering Laboratory for Key Technology of Birth Defect Control and Prevention, Screening and Diagnostic R and D Center, Hangzhou, China

**Keywords:** Monogenic genetic diseases, Newborn screening, Next-generation sequencing, Monogenic genetic diseases, Regional features

## Abstract

**Background:**

Newborn screening (NBS) is an important and successful public health program that helps improve the long-term clinical outcomes of newborns by providing early diagnosis and treatment of certain inborn diseases. The development of next-generation sequencing (NGS) technology provides new opportunities to expand current newborn screening methodologies.

**Methods:**

We designed a a newborn genetic screening (NBGS) panel targeting 135 genes associated with 75 inborn disorders by multiplex PCR combined with NGS. With this panel, a large-scale, multicenter, prospective multidisease analysis was conducted on dried blood spot (DBS) profiles from 21,442 neonates nationwide.

**Results:**

We presented the positive detection rate and carrier frequency of diseases and related variants in different regions; and 168 (0.78%) positive cases were detected. Glucose-6-Phosphate Dehydrogenase deficiency (G6PDD) and phenylketonuria (PKU) had higher prevalence rates, which were significantly different in different regions. The positive detection of *G6PD* variants was quite common in south China, whereas *PAH* variants were most commonly identified in north China. In addition, NBGS identified 3 cases with DUOX2 variants and one with SLC25A13 variants, which were normal in conventional NBS, but were confirmed later as abnormal in repeated biochemical testing after recall. Eighty percent of high-frequency gene carriers and 60% of high-frequency variant carriers had obvious regional differences. On the premise that there was no significant difference in birth weight and gestational age, the biochemical indicators of *SLC22A5* c.1400C > G and *ACADSB* c.1165A > G carriers were significantly different from those of non-carriers.

**Conclusions:**

We demonstrated that NBGS is an effective strategy to identify neonates affected with treatable diseases as a supplement to current NBS methods. Our data also showed that the prevalence of diseases has significant regional characteristics, which provides a theoretical basis for screening diseases in different regions.

**Supplementary Information:**

The online version contains supplementary material available at 10.1007/s12519-022-00670-x.

## Introduction

Newborn screening (NBS), an important and successful public health program, refers to the specific examination of inherited and congenital diseases that seriously threaten the health of newborns in the neonatal period [1, 2]. NBS aims to improve long-term clinical outcomes by providing interventions for the early diagnosis and treatment of these diseases before the onset of symptoms in affected newborns [3]. Since the start of NBS in 1961, new methods have been continuously introduced into NBS, including the bacterial inhibition test for phenylketonuria (PKU) screening [4], the enzyme activity test for galactosemia screening [5], and the radioimmunoassay for congenital hypothyroidism (CH) screening [6]. With the application of tandem mass spectrometry (MS/MS) in the 1990s, it was possible to screen multiple inherited metabolic diseases (IMDs) in a single assay, greatly expanding the screened diseases of NBS [7]. NBS has been widely recognized as an important measure to reduce the morbidity and mortality of neonatal diseases.

At present, MS/MS and other biochemical methodologies are the main screening methods for neonatal IMDs in China [8]. By measuring the levels of amino acids, succinylacetone and acylcarnitines in neonatal dried blood spots (DBS), MS/MS can screen dozens of IMDs through a single experiment, including oxidative metabolic disorders of amino acids, fatty acids and organic acids [9, 10]. However, there are limitations in the current screening technologies, including a limited number of diseases screened, missing detection of newborns with variable biochemical changes at the time of screening, difficulty in interpreting results, and the possibility of false-negative and false-positive screening results [11, 12].

Next-generation sequencing (NGS) is a high-throughput parallel sequencing technology that can analyze the sequences of millions of DNA molecules simultaneously at much lower cost and higher speed than Sanger sequencing [13]. Since the introduction of NGS, it has been quickly and widely adopted in both research and clinical applications. NGS makes it possible to analyze the whole human genome (whole genome sequencing, WGS) or the coding regions of all genes (whole exome sequencing, WES) at an affordable cost [14]. NGS is now widely used in the screening of neonatal genetic disorders [15]. NGS could expand the screening of genetic diseases and facilitate the early detection of genetic defects [16]. Furthermore, the application of NGS in NBS could clarify the variation source and types of genetic disorders from the molecular perspective, provide a basis for genetic counseling, and improve the clinical outcome of children [17]. In the USA, the newborn sequencing in genomics medicine and public health (NSIHT) consortium, funded by the National Institutes of Health (NIH), was established [18]. The BabySeq project, a part of the NSIHT project, was a pilot randomized clinical trial based on WES, which aimed to explore the utility of NGS in genetic screening in healthy and sick newborns and compare the clinical impacts of NGS and routine neonatal screening [19, 20]. A recent study published by the BabySeq project displayed results of risk of childhood onset, carrier status, risk of operable adult-onset disease, and pharmacogenomics from NGS of 159 newborns [21]. However, NGS application in NBS is still in its early infancy, and most NGS application modes involve sequencing positive or suspected children for biochemical screening [22]. In China, a few studies explored genetic screening in newborns, such as hearing loss and other neonatal diseases [23, 24]. Nevertheless, our understanding and experiences of implementing newborn genetic screening of multiple diseases are limited.

In the current study, a large-scale, multicenter prospective analysis was conducted to screen multiple genetic diseases from DBS profiles of 21,442 neonates with a customized newborn genetic screening (NBGS) panel, which has been used in a previous retrospective study [25]. A total of 75 neonatal inborn disorders and 135 genes were carefully selected to be analyzed by the NBGS panel, and the regional incidences and carrier frequencies of selected congenital diseases of these newborns in different regions of China were explored. The screening methods for major genes and pathogenic variants of genetic disorders reported in the current research could improve the detection range of NBGS and contribute to genetic counseling and clinical communication.

## Methods

### Study subjects

A total of 21,442 newborn samples were randomly collected from November 2020 to November 2021. The samples were collected by 12 hospitals from 6 regions, including 1907 samples from Maternal and Child Health Care Hospital of Shandong Province (SDH), 1990 samples from Jinan Maternal and Child Health Care Hospital (JNH), 2050 samples from Maternal and Child Health Care Hospital of Shanxi Province (SXH), 904 samples from Changzhi Maternal and Child Health Care Hospital (CZH), 2060 samples from Ningbo Women and Children’s Hospital (NBH), 1999 samples from Shenzhen Hospital Affiliated to University of Chinese Academy of Sciences (SZH), 1789 samples from Maternal and Child Health Care Hospital of Xinjiang Uygur Autonomous Region (XJH), 1837 samples from Maternal and Child Health Care Hospital of Ningxia Hui Autonomous Region (NXH), 2019 samples from Changsha Maternal and Child Health Care Hospital (CSH), 1874 samples from Chongqing Maternal and Child Health Care Hospital (CQH), 2013 samples from Guiyang Maternal and Child Health Care Hospital (GYH), and 1000 samples from Maternal and Child Health Care Hospital of Yunnan Province (YNH). The inclusion criteria of newborns involved in this study were as follows: (1) neonates had undergone or would undergo MS/MS; (2) Chinese singleton newborns; (3) the parents were in good health, without serious acute or chronic medical history and clear genetic diseases, and (4) follow-up to the end of the project. The exclusion criteria were as follows: (1) those who did not meet the inclusion criteria; (2) parents were not Chinese; (3) the infant was older than 28 days; (4) one of the multiple pregnancies; (5) newborns could not provide a dry blood spot with a diameter greater than 8 mm, and (6) assisted pregnancy (including in *vitro* fertilization and embryo transfer (IVF-ET), intracytoplasmic sperm injection (ICSI) pregnancy) and newborns born after receiving preimplantation genetic screening tests during pregnancy. All the parents of the 21,442 newborns signed informed consent forms. This study was approved by the institutional review board of the ethics committee in all of the above hospitals, and the procedures were in accordance with the seventh revision of the Helsinki Declaration (2013).

### Study design

The 21,442 newborn samples were all subjected to NBGS and conventional NBS (C-NBS). For the NBGS, dried blood spots (4 × 3.2 mm) harvested from the 21,442 samples were screened using an NBGS panel, which includes 1189 amplicons covering 2527 known variants of 135 genes associated with 75 neonatal genetic diseases [25]. For C-NBS, G6PDD screening was performed with the GSP® Neonatal G6PD fluoroimmunoassay kit (PerkinElmer, Finland), time-resolved fluoroimmunoassay (TRFIA) was operated to detect thyroid-stimulating hormone (TSH) for CH screening with a GSP® Neonatal hTSH kit (PerkinElmer), and MS/MS was proceeded to screen IMDs. For clinical profiling, newborn birth weight (in grams) and gestational age (GA, in weeks) were collected from all enrolled newborns. The list of 75 disorders and genes included in the NBGS are shown in Table S1.

### Genetic screening and bioinformatic analysis

Dried blood spots collected from neonates were used to extract genomic DNA via a nucleic acid automatic extraction system (Bioer, China). NGS libraries were generated by amplifying targeted regions with an ultra multiplex PCR system based on the SLIMamp (StemLoop Inhibition Mediated amplification) method [26]. The quality of the libraries was assessed by Bioanalyzer 2100 (Agilent Technologies, Santa Clara, CA, USA). High-throughput sequencing was carried out using an Illumina NextSeq 500 according to the manufacturer’s protocol.

For base calling and raw data generation, bcl2fastq (Illumina) was adopted to process the raw image files. Low-quality sequencing reads were subsequently excluded, and the NCBI human reference genome (hg19/GRCh37) was used to align the remaining reads. The minor allele frequencies (MAFs) of the known variants were identified with the accordance of the 1000 Genome Project, dbSNP and Gnomad. Public and commercial databases, such as OMIM, ClinVar and Human Gene Mutation Database, were used for variant annotations. Bioinformatic tools were implemented for variant interpretation, including SIFT, PolyPhen-2 and MutationTaster, and PROVEAN. The descriptions of these online bioinformatic tools and databases are shown in Table S2.

In the present study, the pathogenicity of the variant was evaluated manually according to the American College of Medical Genetics and Genomics (ACMG) variant interpretation guidelines and updates published by ClinGen. The variations were classified into five categories: pathogenic (P), likely pathogenic (LP), unknown significance (VUS), likely benign (LB) and benign (B). The panel we used included 4 mitochondrial diseases and 131 monogenic diseases, of which monogenic diseases were divided into three groups: (1) dominantly inherited diseases: pathogenic or likely pathogenic (P/LP) variants in genes; (2) recessively inherited diseases: biallelic P/LP variants in genes, and (3) X-linked recessive inheritance and X-linked dominant inheritance.

### Statistical analysis

In the present study, the observational indicators included the ratios of the numbers of detected positive and carriers of the target gene to the number of newborns enrolled in this region, which were the positive rate and carrying frequency of this gene in this region, respectively. In addition, the proportions of the numbers of positive and carrying high-frequency variations in the total detected amount in a region were the positive MAFs and carrying MAFs of this region, respectively. Data were statistically analyzed using SPSS 19.0 (IBM, USA). The difference in a single index among multiple regions was calculated using the chi-square test. *P* < 0.05 was considered a significant difference.

## Results

### Distribution of the screening population and positive detection/carrier frequencies

The screening population included 21,442 newborns, who were divided into six groups according to their region of the enrollment hospital, including North China (*n* = 6851, SDH, JNH, SXH and CZH), Northwest China (*n* = 3626, XJH and NXH), East China (*n* = 2060, NBH), Central China (*n* = 2019, CSH), Southwest China (*n* = 4887, CQH, GYH and YNH), and South China (*n* = 1999, SZH). Positive detection was defined according to the following standards: AR ≥ 2 variants; AD ≥ 1 variant; XLR Male ≥ 1 variant, female ≥ 2 variants or XLD ≥ 1 variant. A carrier is defined according to the following standards: AR = 1 variant or XLR Female = 1. The overall positive detection rates covered by NBGS screening in each region ranged from 0.1% to 0.38% (except *G6PD* variants), the lowest in South China and the highest in North China (Table 1). There was no significant difference in the pathogenic variant carrier frequencies for one variant of each region.

**Table 1:** The overall prevalence and carrier frequencies in different regions (*G6PD* excluded)

Regions	North China	Northwest China	East China	Central China	South China		Southwest China	*P* value
Prevalence (%)	26/6851 (0.38%)	8/3626 (0.22%)	6/2060 (0.29%)	7/2019 (0.35%)	2/1999 (0.1%)	11/4887 (0.23%)		0.300
Pathogenic variant carrier frequencies for 1 variant	1473/6851 (21.50%)	768/3626 (21.18%)	463/2060 (22.48%)	447/2019 (22.14%)	427/1999 (21.36%)	11/4887 (0.23%)		0.272
Pathogenic variant carrier frequencies for 2 variants	241/6851 (3.52%)	81/3626 (2.23%)	81/2060 (3.93%)	72/2019 (3.57%)	66/1999 (3.30%)	1127/4887 (23.06%)		0.003
Pathogenic variant carrier frequencies for 3 or more variants	38/6851 (0.55%)	14/3626 (0.39%)	11/2060 (0.53%)	7/2019 (0.35%)	30/1999 (1.50%)	150/4887 (3.07%)		< 0.001

### Regional features of positive detection rates

**Fig. 1:**
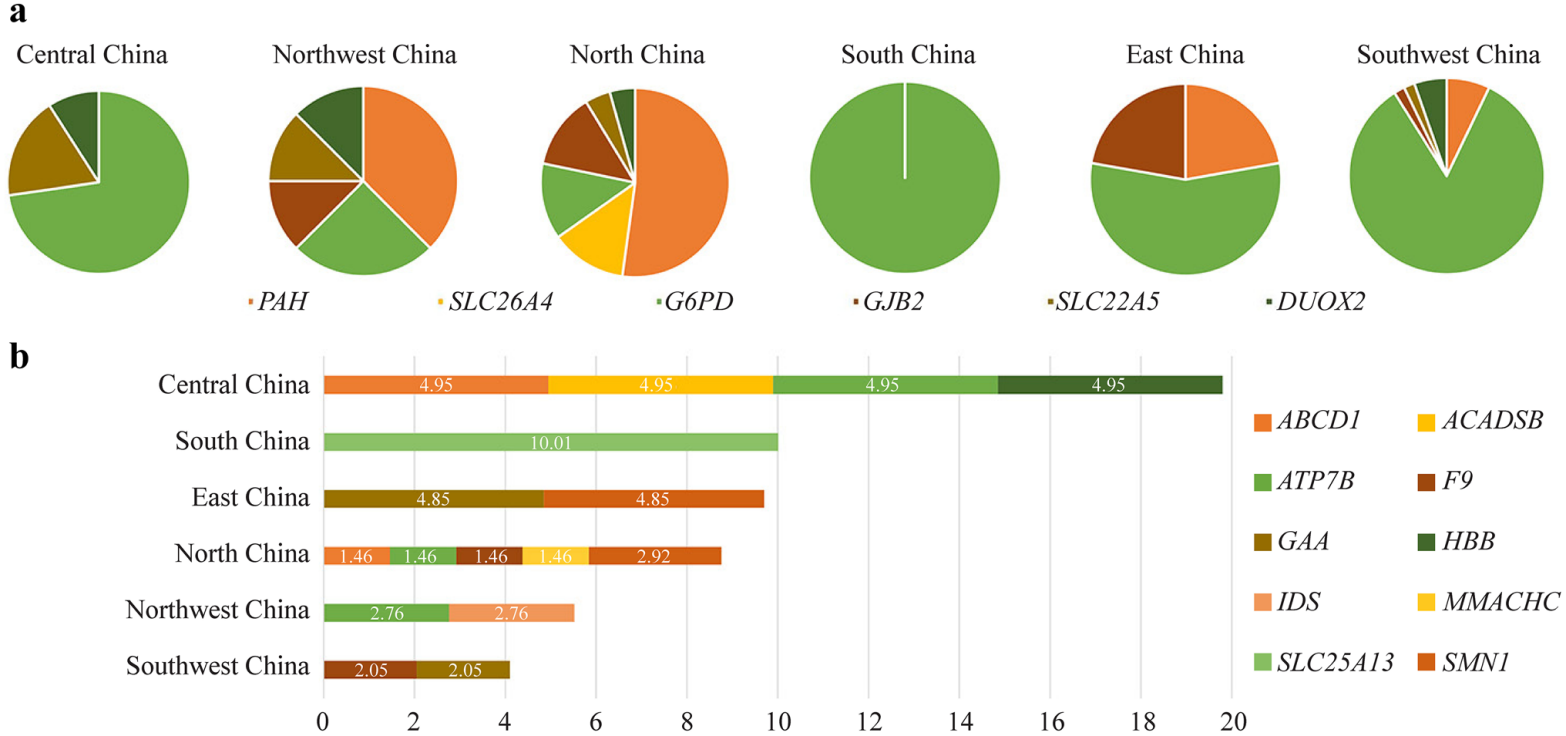
Distribution of gene variation positive rates by subgroups. **a** The fractions of the top 6 common gene variations in each geological subgroup. **b** The positive rates of the remaining 10 most common gene variations in each subgroup indicated as 1 in 10,000

**Fig. 2:**
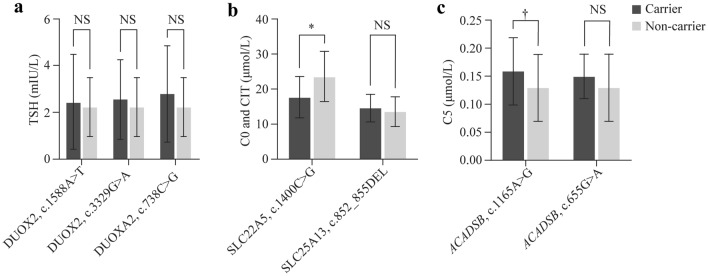
Correlation of genotype and biochemical indicators. **a** The difference in thyroid-stimulating hormone (TSH) between carriers of *DUOX2* variants and non-carriers; **b** The difference in C0 (free carnitine) and CIT (citrulline) between carriers of *SLC22A5* and *SLC25A13* variants and non-carriers; **c** The difference in C5 (C5 acylcarnitine) between carriers of *ACADSB* variants and non-carriers

Several regional features of positive detection rates were observed. When a hemizygous variant in an X-linked gene, or biallelic variants in a autosomal recessive gene, were detected, the subject is considered as a positive case. In the whole cohort, the top 6 genes with the most positive cases were glucose-6-phosphate dehydrogenase (*G6PD*) (50.37 in 10,000), phenylalanine hydroxylase (*PAH*) (9.79 in 10,000), gap junction beta 2 (*GJB2*) (3.26 in 10,000), dual oxidase 2 (*DUOX2*) (2.80 in 10,000), solute carrier family 22 member (*SLC22A5*) (2.33 in 10,000), and solute carrier family 26 member 4 (*SLC26A4*) (1.40 in 10,000) (Fig. 1a). The X-linked incomplete dominant *G6PD* variations were quite common in South China but relatively rare in North China (Table 2), whereas *PAH* variations were most commonly identified in North China (Table S3). Overall positive detection rates were similar in Southwest and Northwest China and were not detected in Central China and South China. The positive rates of other target genes are shown in Fig. 2b, which had large geographical differences.

**Table 2:** The positive detection rate of *G6PD* gene variants of different regions. ^a^The detection rates were 1 in 10,000

*G6PD* gene variants^a^	c.1388G > A	c.1376G > T	c.95A > G	c.1024C > T	c.871G > A	c.392G > T	c.406C > T	c.487G > A	c.1004C > A	c.844G > C	c.517 T > C	Total
North China	0.97	0	0	0.97	0	0	0.97	0	0	0	0	2.92
Northwest	0	0	0	1.84	0	0	0	0	0	1.84	0	3.68
South China	36.69	66.7	13.34	6.67	10.01	10.01	0	0	3.34	0	0	146.74
East China	3.24	3.24	0	0	6.47	0	0	3.24	0	0	0	16.18
Southwest	23.19	12.28	10.91	8.18	0	4.09	5.46	1.36	1.36	0	1.36	68.21
Central China	9.91	3.3	6.6	6.6	3.3	0	0	0	0	0	0	29.72

A total of 11 different *G6PD* pathogenic variants were observed in the current study, and their positive rates are presented in Table 2. Among them, c.1388G > A, c.1376G > T, c.95A > G, and c.1024C > T were the four pathogenic variants observed frequently. The frequency of *G6PD* gene pathogenic variants varied in different regions (*P* value < 0.001). A group of 108 positive cases of *G6PD* variations confirmed in newborns were detected by NBGS. *G6PD* variants were detected in 5 females, including 4 compound heterozygous variants and 1 homozygous variant, and the rest were males. The positive rate of *G6PD* detected by NBGS was 0.50% (108/21,442). After the *G6PD* enzyme activity test, the positive rate was confirmed to be 94.44% (102/108), no feedback was confirmed to be 1.85% (2/108), and normal results were confirmed to be 3.70% (4/108). The distribution characteristics of *G6PD* pathogenic variant frequency showed a decreasing trend from south to north in China. Among them, south China was the highest (146.74 in 10,000), followed by Southwest (68.21 in 10,000), Central China (29.72 in 10,000), East China (16.18 in 10,000), and Northwest (3.68 in 10,000), and North China (2.92 in 10,000) was the lowest.

All *PAH* pathogenic variants detected and their positive rates are presented in Table S3. The distribution of PKU among the Chinese population showed geographical differences (*P*  < 0.001). North China had a spectrum of 18 distinct *PAH* gene variants, which was the region with the most variants in China. After additional biochemical analysis, the positive rate was confirmed to be 52.38% (11/21). After family verification, variants located on the same chromosome were detected in two cases, which can be considered carriers. Eight patients were lost to follow-up. c.158G > A was the most prevalent variant (MAF: 1.17 in 10,000). Currently, all compound heterozygous variants with c.158G > A have normal clinical phenotypes.

### Distribution of frequent pathogenic gene and variant carrier frequencies in different regions

The top 10 most frequent pathogenic gene carrier frequencies are presented in Table 3. Among them, *DUOX2*, *PAH*, *GJB2*, ATPase copper transporting beta (*ATP7B*) and *SLC26A4* were the five pathogenic gene carrier frequencies most frequently observed. Except for *GJB2*, the pathogenic gene carrier frequencies of the other genes were significantly different in different regions. Seventy percent of high-frequency carrier genes correspond to high-frequency carrier variants.

**Table 3:** The distribution of frequent pathogenic gene carrier frequencies in different regions

Region	North China	Northwest	East China	Central China	South China	Southwest	*P* value
*DUOX2*	153/6851 (2.23%)	76/3626 (2.10%)	81/2060 (3.93%)	86/2019 (4.26%)	63/1999 (3.15%)	193/4887 (3.95%)	< 0.001
*PAH*	225/6851 (3.28%)	136/3626 (3.75%)	43/2060 (2.09%)	33/2019 (1.63%)	41/1999 (2.05%)	83/4887 (1.70%)	< 0.001
*GJB2*	185/6851 (2.70%)	88/3626 (2.43%)	48/2060 (2.33%)	52/2019 (2.58%)	40/1999 (2.00%)	106/4887 (2.17%)	0.375
*ATP7B*	132/6851 (1.93%)	64/3626 (1.77%)	51/2060 (2.48%)	41/2019 (2.03%)	46/1999 (2.30%)	144/4887 (2.95%)	0.002
*SLC26A4*	177/6851 (2.58%)	73/3626 (2.01%)	49/2060 (2.38%)	35/2019 (1.73%)	41/1999 (2.05%)	55/4887 (1.13%)	< 0.001
*SLC22A5*	99/6851 (1.45%)	45/3626 (1.24%)	43/2060 (2.09%)	52/2019 (2.58%)	30/1999 (1.50%)	104/4887 (2.13%)	< 0.001
*MMACHC*	176/6851 (2.57%)	43/3626 (1.19%)	19/2060 (0.92%)	17/2019 (0.84%)	16/1999 (0.80%)	59/4887 (1.21%)	< 0.001
*ACADSB*	39/6851 (0.42%)	23/3626 (0.63%)	19/2060 (0.92%)	32/2019 (1.58%)	34/1999 (1.70%)	121/4887 (2.48%)	< 0.001
*SLC25A13*	59/6851 (0.86%)	33/3626 (0.91%)	26/2060 (1.26%)	36/2019 (1.78%)	29/1999 (1.45%)	69/4887 (1.41%)	0.003
*ACADS*	72/6851 (1.05%)	39/3626 (1.08%)	22/2060 (1.07%)	22/2019 (1.09%)	27/1999 (1.35%)	37/4887 (0.76%)	0.324

The top 10 most frequent pathogenic variant carrier frequencies are presented in Table 4. Among them, *DUOX2* c.1588A > T, *GJB2* c.235del, *SLC26A4* c.919-2A > G, *SLC22A5* c.1400C > G, and solute carrier family 25 member 13 (*SLC25A13*) c.852_855del were the five pathogenic variants most frequently observed. *DUOX2* c.1588A > T, *SLC26A4* c.919-2A > G, *SLC22A5* c.1400C > G, *SLC25A13* c.852_855del, *DUOX2* c.3329G > A, and acyl-CoA dehydrogenase short/branched chain (*ACADSB*) c.1165A > G had significant regional differences.

**Table 4:** The distribution of frequent pathogenic variants carrier frequencies in different regions

Region	North China	Northwest China	East China	Central China	South China	Southwest China	*P* value
*DUOX2* c.1588A > T	66/13,702 (0.48%)	45/7,252 (0.62%)	38/4,120 (0.92%)	66/4,038 (1.63%)	46/3,998 (1.15%)	125/9,774 (1.28%)	< 0.001
*GJB2* c.235del	127/13,702(0.93%)	50/7252 (0.69%)	37/4120 (0.90%)	38/4038 (0.94%)	28/3998 (0.70%)	74/9774 (0.76%)	0.346
*SLC26A4* c.919-2A > G	104/13,702 (0.76%)	33/7252 (0.46%)	24/4120 (0.58%)	20/4038 (0.50%)	19/3998 (0.48%)	33/9774 (0.34%)	0.001
*SLC22A5* c.1400C > G	73/13,702 (0.53%)	33/7252 (0.46%)	21/4120 (0.51%)	16/4038 (0.40%)	5/3998 (0.13%)	40/9774 (0.41%)	0.027
*SLC25A13* c.852_855del	27/13,702 (0.20%)	12/7252 (0.17%)	16/4120 (0.39%)	28/4038 (0.69%)	20/3998 (0.50%)	52/9774 (0.53%)	< 0.001
*DUOX2* c.3329G > A	44/13,702 (0.32%)	12/7252 (0.17%)	32/4120 (0.77%)	8/4038 (0.20%)	9/3998 (0.23%)	28/9774 (0.29%)	< 0.001
*DUOXA2* c.738C > G	49/13,702 (0.36%)	16/7252 (0.22%)	6/4120 (0.15%)	12/4038 (0.30%)	7/3998 (0.18%)	35/9774 (0.36%)	0.085
*ACADSB* c.1165A > G	0/13,702 (0%)	1/7252 (0.01%)	4/4120 (0.10%)	20/4038 (0.50%)	16/3998 (0.40%)	79/9774 (0.81%)	< 0.001
*ATP7B* c.2333G > T	45/13,702 (0.33%)	16/7252 (0.22%)	13/4120 (0.32%)	8/4038 (0.20%)	4/3998 (0.10%)	23/9774 (0.24%)	0.141
*ACADSB* c.655G > A	26/13,702 (0.19%)	12/7252 (0.17%)	8/4120 (0.19%)	2/4038 (0.05%)	7/3998 (0.18%)	29/9774 (0.30%)	0.076

### Findings pertaining to monogenic-disease risk

Except for G6PDD and PKU, we found other monogenic diseases in the preliminary screening of NBGS, and the results are shown in Table 5. We found seven *GJB2* variant-positive cases (3.26 in 10,000), and all seven cases were verified by Sanger sequencing and clinical confirmation. Six positive cases with *DUOX2* variants (2.80 in 10,000) were found in our study, all of which were confirmed by Sanger family verification and clinical confirmative diagnosis, except for one case that had no available follow-up data. Among the five positive cases of *SLC22A5* variants discovered (2.33 in 10,000), all cases were excluded as negative via Sanger family verification, and no clinical follow-up data were available. Among the three positive cases with *SLC26A4* variants (1.40 in 10,000), two cases were confirmed by Sanger sequencing and clinical evaluation. One patient displayed the appearance of the bilateral enlarged vestibular aqueduct on inner ear MRI, and the other patient had no follow-up data available. Two positive cases with *SLC25A13* variants (0.93 in 10,000) were identified, one of which was ruled out as negative with Sanger sequencing, and the other was confirmed clinically. Furthermore, three positive cases of *ATP7B* variants (1.40 in 10,000) were identified, two cases of which were confirmed by Sanger sequencing and clinically confirmed, whereas no follow-up data were available for the other case. For three positive cases with *SMN1* exon 7 deletions (1.40 in 10,000), copy numbers of *SMN2* were further analyzed. We found that they had two copies and three copies and four copies of *SMN2,* respectively. The baby who had two copies of *SMN2* was admitted in the ICU with lung infection. In addition, two positive alpha glucosidase (*GAA*) variants (0.93 in 10,000) were confirmed by Sanger sequencing. One case with *MMACHC* variants and one with *HBB* variants were identified, both of which were confirmed by Sanger sequencing and additional clinical evaluation. Moreover, all of the screened two positive cases of ATP binding cassette subfamily D member 1 (*ABCD1*) variants (0.93 in 10,000), two positive cases of coagulation factor IX (*F9*) variants (0.93 in 10,000), and one positive case of iduronate 2-sulfatase (*IDS*) (0.47 in 10,000) were confirmed by Sanger sequencing.

**Table 5:** Findings pertaining to monogenic-diseases risk. *Inh* inheritance, *AD* autosomal dominant, *AR* autosomal recessive, *XLR* X-linked recessive, *Hom* homozygote, *Het* heterozygote, *Comp het* compound heterozygote, *Hemi* hemizygote, *M* male, *F* female, *TSH* thyroid-stimulating hormone, *C0* free carnitine, *C2/C3/C5*, C2/C3/C5 acylcarnitine, *GAA* acid α-glucosidase. ^a^The ratio unit is 1 in 10,000

Genes	Diseases	Total positive frequency^a^	Inh	Variant(s)	Zygosity	Sex	Phenotype at follow-up
*GJB2*	Non-syndromic hearing loss	7/21,442 (3.26)	AD	c.176_191del, c.235del, c.508_511dup	Hom/het	M/F	Hearing screening failed for 7 cases
*DUOX2*	Congenital Hypothyroidism	6/21,442 (2.80)	AR	c.596del, c.1588A > T, c.3329G > A, c.602dup	Hom/het	M/F	TSH increased for 5 cases
*SLC22A5*	Primary carnitine deficiency	5/21,442 (2.33)	AR	c.1400C > G, c.845G > A	Hom/het	M/F	Normal C0 for 5 case
*SLC26A4*	Non-syndromic hearing loss	3/21,442 (1.40)	AR	c.919-2A > G, c.589G > A, c.1975G > C	Het/hom	M/F	Hearing screening failed for 2 cases
*ABCD1*	X-linked adrenoleukodystrophy	2/21,442 (0.93)	XLR	c.1552C > T,c.1415_1416de	Het	F	No follow-up data available
*SLC25A13*	Citrin deficiency	2/21442 (0.93)	AR	c.615 + 5G > A, c.852_855del, c.550C > T, c.1638_1660dup	Het	M	Increased citrulline in 1 case
*ACADSB*	2-Methylbutyryl-CoA dehydrogenase deficiency	1/21,442 (0.47)	AR	c.1165A > G, c.655G > A	Comp het	M	Normal C5 for 1 case
*MMACHC*	Methylmalonic aciduria	1/21,442 (0.47)	AR	c.481C > T, c.80A > G	Het	M	C3 and C3/C2 increased for 1 case
*ATP7B*	Wilson Disease	3/21,442 (1.40)	AR	c.2333G > T, c.1708-5 T > G, c.2975C > T, c.2621C > T, c.3008C > T	Hemi/het	M/F	Ceruloplasmin decreased for 2 cases
*SMN1*	Spinal muscular atrophy	3/21442 (1.40)	AR	/	Hom	M/F	Admitted in ICU for 1 case
*GAA*	Glycogen storage disease	2/21442 (0.93)	AR	c.1933G > A, c.752C > T, c.761C > T, c.1942G > A	Het	M	Decreased GAA in 1 case
*F9*	Hemophilia B	2/21442 (0.93)	XLR	c.838 + 1_838 + 16del, c.224G > A	Het	F	No follow-up data available
*HBB*	Beta-thalassemia	1/21442 (0.47)	AR	c.316-197C > T	Hom	F	β-thalassemia confirmed in 1 case
*IDS*	Mucopolysaccharidosis	1/21442 (0.47)	XLR	c.998C > T	Hemi	M	No follow-up data available

### Correlation in biochemical indicators between carriers and non-carriers

In this study, east China, northwest China, and southwest China were selected to analyze the correlation in biochemical indicators. We selected from the top 10 most frequent pathogenic variant carriers. *DUOX2* c.1588A > T, *SLC22A5* c.1400C > G, *SLC25A13* c.852_855del, *DUOX2* c.3329G > A, *DUOXA2* c.738C > G, *ACADSB* c.1165A > G, and *ACADSB* c.655G > A were selected, corresponding to the biochemical indicators thyroid-stimulating hormone (TSH) (Fig. 2a), free carnitine (C0), citrulline (CIT) (Fig. 2b), and methylcrotonyl carnitine (C5) (Fig. 2C), excluding the deafness-related *GJB2* and *SLC26A4* and the non-C-NBS gene *ATP7B*. Only single-variation samples were selected for biochemical indicator analysis. There were no significant differences in birth weight or gestational age between the variant carriers and non-carriers in the three regions (Table S4). Although the indices were all within the normal range, the C0 index of *SLC22A5* c.1400C > G carriers was significantly lower than that of non-carriers (control group), while the C5 index of *ACADSB* c.1165A > G carriers was significantly higher than that of controls.

## Discussion

Newborn disease screening is one of the important measures for the three-level prevention of birth defects, which could prevent serious, life-threatening health problems through early intervention [27]. At present, NC NEXUS [28, 29] and Babyseq [30, 31] of newborn screening by NGS have been carried out in many places in the United States. Methodological evaluation of genetic screening by applying WES and WGS to a retrospective cohort analysis [28, 30, 32]. The technical methods we adopted in this study were more advanced and easy to operate, which also greatly shortened the reporting cycle and reduced the difficulty of report interpretation when compared to that of NC NEXUS and Babyseq. Another study [32] found that NGS technology can be used as a supplement to C-NBS, reducing the false-positive rate of screening results, resolving inconclusive results from C-NBS, and identifying pathogenic variant loci in affected individuals.

**Fig. 3:**
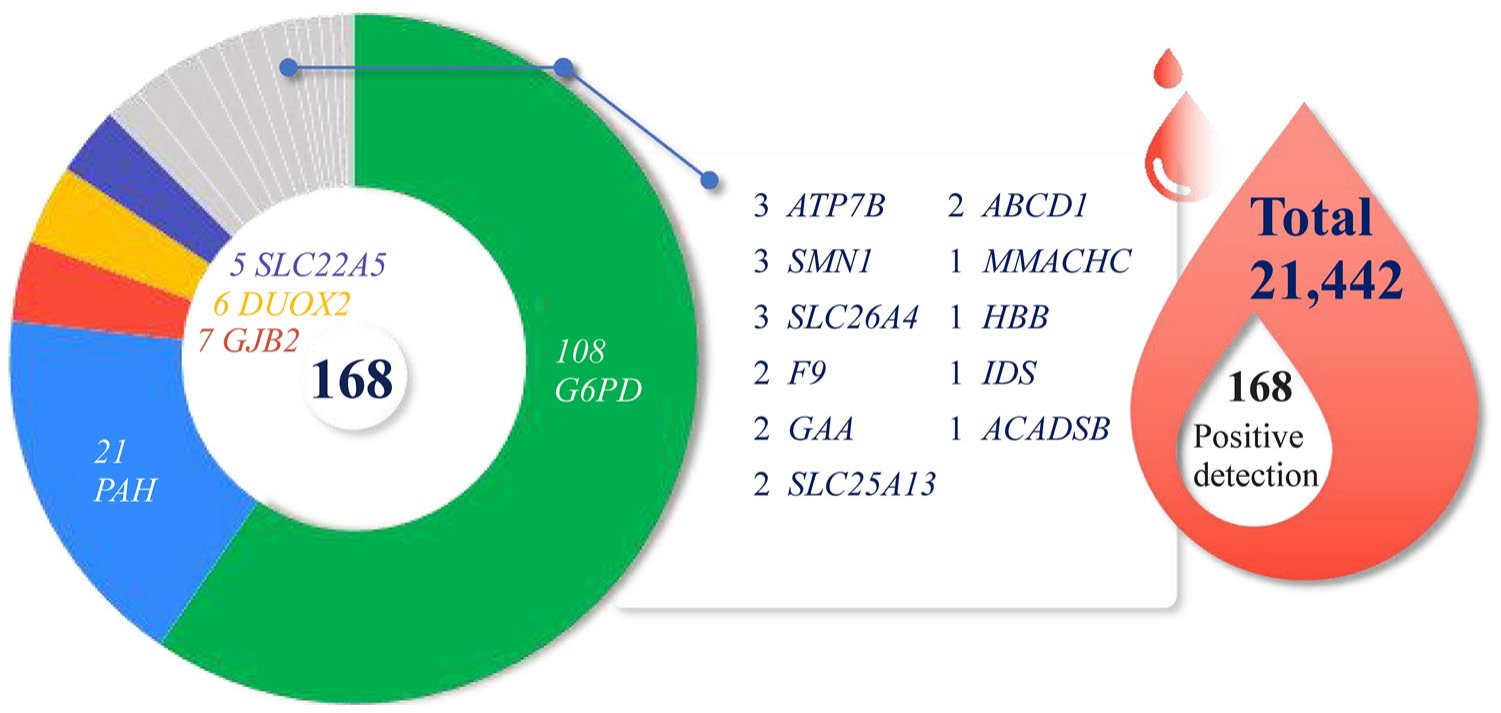
Summary of positively identified neonates and related genes in this study. *G6PD* glucose-6-phosphate dehydrogenase, *PAH* phenylalanine hydroxylase, *GJB2 *gap junction beta 2, *DUOX2* dual oxidase 2, *SLC22A5* solute carrier family 22 member, *SLC26A4* solute carrier family 26 member 4, *GAA *acid α-glucosidase, *ATP7B* ATPase copper transporting beta, *ABCD1* ATP binding cassette subfamily D member 1, *IDS* iduronate 2-sulfatase, *GAA* alpha glucosidase, *F9* coagulation factor IX, *HBB* hemoglobin beta-chain, *SMN1* survival of motor neuron 1

In this study, a large-scale, multicenter prospective analysis of 21,442 neonates was conducted by applying an NGS panel covering 135 genes associated with 75 neonatal inborn disorders. The study was performed using simple-to-operate and customizable multiplex PCR amplicon sequencing technology [33]. We present the positive and carrier frequencies of gene variations in different regions, illustrating the regional features in China. In our study, from these 21,442 infants, pathogenic variations were detected in 5700 infants. Among the 5700 infants, 168 cases were positive, and 5532 were pathogenic gene carriers. The 168 (0.78%) positive cases were detected by NBGS (Fig. 3), of which 164 cases were verified by Sanger family verification, and 4 were lost to follow-up. Among the 164 Sanger family verification cases, 7 were excluded as carriers because two pathogenic variants were located on the same chromosome. In addition, there were 149 clinical follow-up cases, of which 135 were confirmed, 7 had normal clinical phenotypes, and 7 were undetected. Among them, 3 cases with *DUOX2* variants and 1 with *SLC25A13* variants were normal in the initial clinical screening. The variants were detected by NBGS, and the clinical diagnosis was confirmed after recall examination.

There was no significant difference in the prevalence of 75 diseases detected by the NBGS panel in different regions. The same diseases, such as G6PDD and PKU, with higher incidences have significant differences in different regions [34, 35]. Among them, the prevalence of G6PDD in South China (2.15%, 43/1,999) and Southwest China (0.96%, 47/4,887) was the highest, and North China (0.04%, 3/6,851) and Northwest China (0.06%, 2/3,626) were the lowest, showing significant geographical differences, which was consistent with existing research [34]. The prevalence of PKU detected by the NBGS was 0.10% (21/21,442), with the highest prevalence in northern China. In addition, three deafness-related gene *GJB3* (gap junction protein beta 3), *MT-RNR1* (mitochondrially encoded 12S rRNA), and *MTTL1* (mitochondrially encoded tRNA leucine 1) variants, excluded in the positive reports due to controversial genotype–phenotype correlation, also had higher prevalences of 0.34% (72 in 21,442), 0.23% (49 in 21,442) and 0.10% (22 in 21,442), respectively. The pathogenicity of *GJB3* variants is believed to lead to delayed deafness [24, 36]. *MT-RNR1* and *MTTL1* variants lead to mitochondrial hearing loss, which has variable penetrance and severity, even within families [37].

The results showed that the top 10 most frequent pathogenic gene and variant carrier frequencies were presented. Seventy percent of high-frequency carrier genes correspond to high-frequency carrier variants. Eighty percent of high-frequency gene carriers and 60% of high-frequency variant carriers had obvious regional differences. On the premise that there was no significant difference in birth weight and gestational age, although the biochemical indicators of *SLC22A5* c.1400C > G and *ACADSB* c.1165A > G carriers were within the normal range, they were significantly different from non-carriers. This shows that the variation in these two sites has a certain influence on the enzyme activity. In addition, we also found 13 cases of chromosomal abnormalities by multiplex PCR, of which 3 were recalled and confirmed, Klinefelter syndrome (XXY) in 2, and XX male syndrome in 1.

In this study, we explored whether the prevalence of diseases has significant regional characteristics, which provides a theoretical basis for screening diseases in different regions. However, our data only represented the neonatal genetic disease prevalence of 12 representative hospitals in the 6 regions. The positive detection rates of the panel were estimated by variant carrier frequencies. This total was composed of monogenetic diseases (41.55 in 10,000), consisting of autosomal dominant (11.36 in 10,000) and X-linked recessive disorders (30.18 in 10,000). Genetic screening could save huge medical costs. Multiplex PCR technology also has the advantages of a short reporting time, easy genetic interpretation, and low cost, which costs only 1/5 of WES.

In summary, we evaluated the incidence and carrier frequencies of 75 neonatal inborn disorders and 135 genes in 21,442 newborns from different regions of China through an NBGS panel. We found that the positive detection and carrier frequency of neonatal inborn disorders in different regions were significantly different. These findings proved that NBGS was a potential strategy for NBS and served as a supplemental tool for C-NBS methods. In addition, our data provide a theoretical basis for screening neonatal inborn disorders in different regions.

## Supplementary Information

Below is the link to the electronic supplementary material.

Supplementary file1 (DOCX 24 KB)

## Data Availability

All data generated or analyzed during this study are included in this published article and supplementary information files.
