# Supplementary material for: A multicenter prospective study of next-generation sequencing-based newborn screening for monogenic genetic diseases in China
Source: World J Pediatr. 2023 Feb 27;19(7):663–73. doi: 10.1007/s12519-022-00670-x (PMC10258179; doi:10.1007/s12519-022-00670-x)
Supplement: Supplementary file 1 — Supplementary file1 (DOCX 24 KB) [file 12519_2022_670_MOESM1_ESM.docx]

*Table S1*. The list of 75 disorders and genes included in NBGS

| NO. | Diseases |  |
| --- | --- | --- |
| **C-NBS screened disorders** | | **Gene** |
| 1 | Methylmalonic aciduria, MMA | *MMAA*  *MMAB*  *MMUT*  *MMADHC*  *MMACHC*  *LMBRD1*  *ABCD4*  *HCFC1* |
| 2 | Propionic acidemia, PA | *PCCA*  *PCCB* |
| 3 | Glutaric acidemia I, GA1 | *GCDH* |
| 4 | Holocarboxylase synthetase deficiency | *HLCS* |
| 5 | Biotinidase deficiency | *BTD* |
| 6 | 3-Hydroxy-3-Methylglutaryl-CoA Lyase deficiency, HMGCLD | *HMGCL* |
| 7 | 3-Hydroxy-3-Methylglutaryl-CoA synthase-2 deficiency, HMGCS2D | *HMGCS2* |
| 8 | Isovaleric acidemia, IVA | *IVD* |
| 9 | 3-Methylcrotonyl-coenzyme A carboxylase deficiency, MCCD | *MCCC1*  *MCCC2* |
| 10 | Malonyl-CoA decarboxylase deficiency, MCD deficiency | *MLYCD* |
| 11 | 3-methylglutaconic aciduria type I, MGCA1 | *AUH* |
| 12 | Ethylmalonic encephalopathy, EE | *ETHE1* |
| 13 | Maple syrup urine disease, MSUD | *BCKDHA*  *BCKDHB*  *DBT*  *DLD* |
| 14 | Tyrosinemia | *FAH*  *TAT*  *HPD* |
| 15 | Hyperphenylalaninemia, HPA | *PAH*  *PCBD1*  *PTS*  *QDPR*  *GCH1*  *SPR* |
| 16 | Hyperhomocysteinemia, HCY | *CBS*  *MTHFR*  *MTRR*  *MTR* |
| 17 | Non-ketotic hyperglycinemia, NKH | *GLDC* |
| 18 | Hypermethioninemia, H-MET | *MAT1A* |
| 19 | Argininemia | *ARG1* |
| 20 | Argininosuccinic aciduria, ASA | *ASL* |
| 21 | Carbamoyl phosphate syntetase I deficiency, CPS1D | *CPS1* |
| 22 | Ornithine transcarbamylase deficiency, OTCD | *OTC* |
| 23 | Citrullinemia type Ⅰ, CTLN1 | *ASS1* |
| 24 | Citrin deficiency, CD | *SLC25A13* |
| 25 | Hyperornithinemia-hyperammonemia-homocitrullinemia syndrome, HHHS | *SLC25A15* |
| 26 | Ornithine aminotransferase deficiency | *OAT* |
| 27 | Very long-chain acyl-CoA dehydrogenase deficiency, VLCAD | *ACADVL* |
| 28 | Long-chain 3-hydroxyl-CoA dehydrogenase deficiency, LCHAD | *HADHA*  *HADHB* |
| 29 | Short chain 3-hydroxyacyl-CoA dehydrogenase deficiency, SCHADD | *HADH* |
| 30 | Medium chain acyl-CoA dehydrogenase deficiency, MCADD | *ACADM* |
| 31 | Short-chain acyl-CoA dehydrogenase deficiency, SCADD | *ACADS* |
| 32 | Glutaric acidemia II, GA2 | *ETFA*  *ETFB*  *ETFDH* |
| 33 | Beta-ketothiolase deficiency, BKD | *ACAT1* |
| 34 | Carnitine palmitoyltransferase II deficiency, CPTII deficiency | *CPT2* |
| 35 | Primary carnitine deficiency, PCD | *SLC22A5* |
| 36 | Carnitine palmitoyltransferase I deficiency, CPT1 deficiency | *CPT1A* |
| 37 | Carnitine-acylcarnitine translocase deficiency, CACT deficiency | *SLC25A20* |
| 38 | 2-methylbutyryl-CoA dehydrogenase deficiency | *ACADSB* |
| 39 | Glucose-6-phosphate dehydrogenase deficiency, G6PD | *G6PD* |
| 40 | Congenital Hypothyroidism, CH | *PAX8*  *THRA*  *THRB*  *TSHB*  *TSHR*  *TG*  *TPO*  *DUOXA2*  *DUOX2* |
| 41 | Non-syndromic hearing loss, NSHL | *GJB2*  *GJB3*  *SLC26A4*  *MT-RNR1*  *MT-TH* |
| **Non C-NBS screened disorders** | |  |
| 42 | N-acetylglutamate synthase deficiency, NAGSD | *NAGS* |
| 43 | Glycogen storage disease, GSD | *G6PC*  *SLC37A4*  *GAA*  *AGL*  *PYGL* |
| 44 | Galactosemia | *GALT*  *GALK1*  *GALE* |
| 45 | Hereditary fructose intolerance, HFI | *ALDOB* |
| 46 | X-linked adrenoleukodystrophy, X-ALD | *ABCD1* |
| 47 | Mucopolysaccharidosis, MPS | *IDUA*  *IDS*  *GALNS*  *NAGLU* |
| 48 | Krabbe Disease, KRB | *GALC* |
| 49 | Gaucher disease, GD | *GBA* |
| 50 | Fabry disease | *GLA* |
| 51 | Niemann-Pick disease | *NPC1*  *NPC2*  *SMPD1* |
| 52 | Beta-thalassemia | *HBB* |
| 53 | Hemophilia B, HB | *F9* |
| 54 | Growth hormone deficiency, GHD | *GH1*  *GHRHR* |
| 55 | Vitamin D-dependent rickets,VDDR | *CYP27B1*  *VDR* |
| 56 | X-linked dominant hypophosphatemia, XLH |  |
| 57 | Cartilage-hair hypoplasia, CHH | *RMRP* |
| 58 | Spinal muscular atrophy, SMA | *SMN1* |
| 59 | Crigler-najjar syndrome, CN | *UGT1A1* |
| 60 | Progressive familial intrahepatic cholestasis, PFIC | *ATP8B1*  *ABCB11* |
| 61 | Wilms tumor, WT1 | *WT1* |
| 62 | Retinoblastoma, RB1 | *RB1* |
| 63 | Mitochondrial encephalomyopathy, lactic acidosis and stroke-like episodes, MELAS | *MT-TL1*  *MT-ND5* |
| 64 | Severe combined immune deficiency, SCID | *ADA*  *IL2RG*  *IL7R*  *JAK3*  *RAG1*  *RAG2*  *BCL11B* |
| 65 | Wilson Disease,WD | *ATP7B* |
| 66 | Congenital bile acid synthesis defect type 1, CBAS1 | *HSD3B7* |
| 67 | X-linked immunodysregulation, polyendocrinopathy, and enteropathy, IPEX | *FOXP3* |
| 68 | X-linked dominant hypophosphatemic rickets | *PHEX* |
| 69 | X-linked recessive chronic granulomatous disease, CGD X | *CYBB* |
| 70 | X-linked agammaglobulinemia, XLA | *BTK* |
| 71 | Wiskott-Aldrich syndrome, WAS | *WAS* |
| 72 | Dihydropyrimidine dehydrogenase deficiency | *DPYD* |
| 73 | Thiopurine toxicity, THPM | *TPMT*  *NUDT15* |
| 74 | Congenial disorders of glycosylation, CDG1A | *PMM2* |
| 75 | Sitosterolemia, STSL | *ABCG5*  *ABCG8* |

*Table S2.* The descriptions of online bioinformatic tools and databases

| Online Bioinformatic Tools | Websites |
| --- | --- |
| *dbSNP* | *http://www.ncbi.nlm.nih.gov/snp* |
| 1000 Genome Project | *http://browser.1000genomes.org* |
| ExAC | *http://exac.broadinstitute.org/* |
| OMIM | *http://www.omim.org* |
| ClinVar | *<http://www.ncbi.nlm.nih.gov/clinvar>* |
| Human Gene Mutation Database | *http://www.hgmd.org* |
| SIFT | *http://sift.jcvi.org* |
| MutationTaster | *http://www.mutationtaster.org* |
| PolyPhen-2 | *http://genetics.bwh.harvard.edu/pph2* |
| PROVEAN | *http://provean.jcvi.org/index.php* |

*Table S3*. The positive detection rates of *PAH* variants of different regions

| **Region^a^** | ***PAH* gene variants** |
| --- | --- |
| Central China (0) |  |
| East China (9.71) | c.727C>T (2.43), c.898G>T (2.43), c.158G>A (2.43), c.157C>T (2.43) |
| North China (17.52) | c.158G>A (2.19), c.782G>A (1.46), c.1238G>C (1.46), c.728G>A (1.46), c.1262T>C (0.73), c.755G>A (0.73), c.688G>A (0.73), c.1197A>T (0.73), c.194T>G (0.73), c.510T>A (0.73), c.1315+6T>A (0.73), c.1316-2A>C (0.73), c.827T>A (0.73), c.212G>A (0.73), c.442-1G>A (0.73), c.694C>T (0.73), c.842+2T>A (0.73), c.516G>T (0.73) |
| Northwest (8.27) | c.898G>T (1.38), c.464G>A (1.38), c.311C>A (1.38), c.1316-1G>A (1.38), c.688G>A (1.38), c.158G>A (1.38) |
| South China (0) |  |
| Southwest (8.18) | c.516G>T (3.07), c.1174T>A (1.02), c.532G>A (1.02), c.728G>A (1.02), c.722del (1.02), c.755G>A (1.02) |

^a^The ratio unit was 1 in 10,000.

*Table S4.* The data of birth weight and gestational age of different regions

| **East China of detection**  **Gender**  Male: 1056 (51.30%)  Female: 1004 (48.70%)  Unknown: 0 (0.00%)  **Birth Weight**  Mean±SD: 3,333.89±394.54  Mediam: 3320  <2,500g: 21 (1.02%)  2,500-4,000g: 1885 (91.50%)  >4,000g: 108 (5.24%)  Unknown: 46 (2.23%)  **Gestational age**  Mean±SD: 39.07±1.03  Mediam: 39.14  <37 weeks: 42 (2.04%)  37-42 weeks: 2016 (97.86%)  >42 weeks: 0 (0.00%)  Unknown: 2 (0.10%) | **East China of incidence and carriers**  **Gender**  Male: 294 (51.31%)  Female: 279 (48.69%)  Unknown: 0 (0.00%)  **Birth Weight**  Mean±SD: 3,320.40±416.16  Mediam: 3300  <2,500g: 3 (0.52%)  2,500-4,000g: 539 (94.07%)  >4,000g: 30 (5.24%)  Unknown: 1 (0.17%)  **Gestational age**  Mean±SD: 39.08±1.03  Mediam: 39.14  <37 weeks: 8 (1.40%)  37-42 weeks: 565 (98.60%)  >42 weeks: 0 (0.00%)  Unknown: 0 (0.00%) | **East China of *DUOX2* carriers**  **Gender**  Male: 48 (59.26%)  Female: 33 (40.74%)  Unknown: 0 (0.00%)  **Birth Weight**  Mean±SD: 3,382.72±452.62  Mediam: 3430  <2,500g: 1 (1.23%)  2,500-4,000g: 73 (90.12%)  >4,000g: 7 (8.64%)  Unknown: 0 (0.00%)  **Gestational age**  Mean±SD: 39.04±1.08  Mediam: 39.14  <37 weeks: 1 (1.23%)  37-42 weeks: 80 (98.77%)  >42 weeks: 0 (0.00%)  Unknown: 0 (0.00%) |
| --- | --- | --- |
| **Northwest of detection**  **Gender**  Male: 1902 (52.45%)  Female: 1719 (47.41%)  Unknown: 5 (0.14%)  **Birth Weight**  Mean±SD: 3,308.85±521.16  Mediam: 3340  <2,500g: 108 (2.98%)  2,500-4,000g: 3421 (94.35%)  >4,000g: 96 (2.65%)  Unknown: 1 (0.03%)  **Gestational age**  Mean±SD: 39.36±1.75  Mediam: 39.57  <37 weeks: 231 (6.37%)  37-42 weeks: 3365 (92.80%)  >42 weeks: 28 (0.77%)  Unknown: 2 (0.06%) | **Northwest of incidence and carriers**  **Gender**  Male: 480 (54.05%)  Female: 406 (45.72%)  Unknown: 2 (0.23%)  **Birth Weight**  Mean±SD: 3,308.94±521.05  Mediam: 3340  <2,500g: 31 (3.49%)  2,500-4,000g: 837 (94.26%)  >4,000g: 20 (2.25%)  Unknown: 0 (0.00%)  **Gestational age**  Mean±SD: 39.34±2.07  Mediam: 39.71  <37 weeks: 54 (6.08%)  37-42 weeks: 824 (92.79%)  >42 weeks: 9 (1.01%)  Unknown: 1 (0.11%) | **Northwest of *PAH* carriers**  **Gender**  Male: 74 (54.41%)  Female: 62 (45.59%)  Unknown: 0 (0.00%)  **Birth Weight**  Mean±SD: 3,305.84±513.13  Mediam: 3362.5  <2,500g: 3 (2.21%)  2,500-4,000g: 130 (95.59%)  >4,000g: 3 (2.21%)  Unknown: 0 (0.00%)  **Gestational age**  Mean±SD: 39.45±1.79  Mediam: 39.71  <37 weeks: 8 (5.88%)  37-42 weeks: 124 (91.18%)  >42 weeks: 4 (2.94%)  Unknown: 0 (0.00%) |
| **Southwest of detection**  **Gender**  Male: 2583 (52.85%)  Female: 2299 (47.04%)  Unknown: 5 (0.10%)  **Birth Weight**  Mean±SD: 3,234.91±426.53  Mediam: 3230  <2,500g: 143 (2.93%)  2,500-4,000g: 4589 (93.90%)  >4,000g: 155 (3.17%)  Unknown: 118 (2.41%)  **Gestational age**  Mean±SD: 38.95±1.31  Mediam: 39  <37 weeks: 197 (4.03%)  37-42 weeks: 4579 (93.70%)  >42 weeks: 3 (0.06%)  Unknown: 108 (2.21%) | **Southwest of incidence and carriers**  **Gender**  Male: 804 (53.89%)  Female: 687 (46.05%)  Unknown: 1 (0.07%)  **Birth Weight**  Mean±SD: 3,238.59±429.65  Mediam: 3250  <2,500g: 51 (3.42%)  2,500-4,000g: 1365 (91.49%)  >4,000g: 49 (3.28%)  Unknown: 27 (1.81%)  **Gestational age**  Mean±SD: 38.95±1.37  Mediam: 39  <37 weeks: 68 (4.56%)  37-42 weeks: 1392 (93.30%)  >42 weeks: 0 (0.00%)  Unknown: 32 (2.14%) | **Southwest of *ATP7B* carriers**  **Gender**  Male: 73 (50%)  Female: 73 (50%)  Unknown: 0 (0.00%)  **Birth Weight**  Mean±SD: 3,238.94±434.44  Mediam: 3250  <2,500g: 6 (4.11%)  2,500-4,000g: 133 (91.10%)  >4,000g: 4 (2.74%)  Unknown: 3 (2.05%)  **Gestational age**  Mean±SD: 38.86±1.67  Mediam: 39  <37 weeks: 9 (6.16%)  37-42 weeks: 134 (91.78%)  >42 weeks: 0 (0.00%)  Unknown: 3 (2.05%) |
